# Cryptococcus neoformans Meningitis in a Patient With Epidermolysis Bullosa and Rheumatoid Arthritis

**DOI:** 10.7759/cureus.103568

**Published:** 2026-02-13

**Authors:** Kay Lin Ng, Karunamuni I Karunaratne

**Affiliations:** 1 General Medicine, Royal Hobart Hospital, Hobart, AUS; 2 Medicine, North West Regional Hospital, Burnie, AUS

**Keywords:** case report, cryptococcal meningitis, epidermolysis bullosa, epidermolysis bullosa acquisita, jak-2 inhibitor, mycophenolate, upadacitinib

## Abstract

Cryptococcal meningitis (CM) is an opportunistic fungal infection with a high mortality rate commonly associated with those with human immunodeficiency virus (HIV) or acquired immunodeficiency syndrome (AIDS). The epidemiology of CM in HIV-negative individuals is unclear, although it is estimated to be increasing in developed countries. We present a case of *Cryptococcus neoformans* meningitis in a 59-year-old HIV-negative female taking long-term mycophenolate (MMF) for epidermolysis bullosa acquisita (EBA) and upadacitinib for rheumatoid arthritis (RA). With an aggressive antifungal regimen, therapeutic lumbar punctures, lumbar drain insertion, and ventriculoperitoneal shunt insertion, the patient's condition improved, and she was discharged from the hospital.

## Introduction

Cryptococcal meningitis (CM) is an opportunistic infection acquired through inhalation of aerosolised particles from bird guano or mopane trees [1,2]. It has a high mortality rate of 19% in those with HIV and AIDS, with around one million cases of cryptococcosis globally and 625,000 deaths each year [3-5].

In 2014, there were approximately 223,100 cases of CM globally, although this number may be larger, as only a few states in the United States of America report their data [3]. In the literature, high-risk patients include those with advanced HIV infections, immune suppression, or solid organ transplantation [1,5]. The exact epidemiology of CM in HIV-negative individuals is unclear, although it is estimated to be increasing in developed countries [4-6]. An aggressive antifungal regimen is required and is often associated with elevated intracranial pressure, requiring repeated therapeutic lumbar punctures or the insertion of drains or shunts [1,3,5,7]. In immunocompetent individuals, cryptococcal invasion is cleared by macrophages and by cytokines such as interferon-γ (IFN-γ), tumour necrosis factor-α (TNF-α), and granulocyte-macrophage colony-stimulating factor (GM-CSF) [8]. For immunocompromised individuals, macrophages and downstream cytokines are suppressed, precluding to cryptococcal infection.

We present a case of *Cryptococcus neoforman*s meningitis in an HIV-negative, transplant-negative patient taking long-term mycophenolate (MMF) for epidermolysis bullosa acquisita (EBA) and upadacitinib for rheumatoid arthritis (RA). There have been some cases of CM reported in those who take MMF and have RA or are taking concurrent long-term steroids with biologics or disease-modifying anti-rheumatic drugs (DMARDs). There are now new reports of CM in those who take JAK inhibitors. However, there have been no reports associated with EBA nor any case reports with the individual taking a combination of MMF and upadacitinib. This case further highlights a case of CM in a patient from a developing country who does not have HIV, does not have a solid organ transplant, and is on combined cumulative immunosuppression with MMF and JAK inhibitors.

## Case presentation

A 59-year-old Caucasian woman living in rural northwest Tasmania presented with a two-week history of worsening headache preceded by a respiratory illness and a three-day history of subjective fevers and confusion, such as becoming disoriented, difficulty speaking, urinary incontinence, childlike behaviour, reduced appetite, and mildly impaired gait.

Relevant medical history included EBA, diagnosed approximately 15 years ago. She has been on courses of immunosuppressive therapies, including topical steroids (clobetasol and betamethasone dipropionate), one year of intravenous immunoglobulin (IVIG) in 2013-2014, azathioprine, dapsone, rituximab, and mycophenolate. In addition to this, she was treated for her seronegative RA and was previously on methotrexate, leflunomide, and hydroxychloroquine. At the time of presentation, she had been taking mycophenolate 1000 mg twice daily for 10 years for EBA and upadacitinib 15 mg daily for RA for five years. Complications related to this immunosuppressive history include febrile neutropenia secondary to infusion port sepsis with *Staphylococcus lentus*-positive blood cultures in 2013, Herpes simplex virus (HSV) keratitis in the right eye, and left-sided *Cytomegalovirus* (CMV) retinitis occurring a year prior to presentation.

On examination, there was drowsiness and a Glasgow Coma Scale of 14 (E4V4M6). There was no other focal neurology. Blood tests showed a C-reactive protein (CRP) level of 20 (normal range: <5 mg/L), troponin I of 25 (normal: <16 ng/L), sodium of 130 (normal: 135-145 mmol/L), potassium of 2.5 (normal: 3.5-5.5 mmol/L), estimated glomerular filtration rate of 78 (normal: >89 mL/min/1.73 m^2^), white cell count of 1.3 (normal: 4.0-11.0/nL), neutrophils of 0.2 (normal: 2.0-7.5/nL), and lymphocytes of 0.5 (normal: 1.0-4.0/nL) (Table 1).

**Table 1:** Blood Investigations H: abnormally high result, L: abnormally low result, ALP: alkaline phosphatase, GGT: gamma glutamyl transferase, ALT: alanine aminotransferase, HCT: hematocrit, MCV: mean corpuscular volume, WBC: white blood cell, eGFR: estimated glomerular filtration rate, nRBC: nucleated red blood cells, CRP: C-reactive protein

Parameters	Day 1 (22/5/25)	Day 2 (23/5/25)	Day 3 (24/5/25)	Day 4 (25/5/25)	Day 5 (26/5/25)	Day 6 (27/5/25)	Units	Reference Range
Sodium	130 (L)	132 (L)	138	141	131 (L)	133 (L)	mmol/L	135–145
Potassium	2.6 (L)	3.7	3.4 (L)	3.5	3.0 (L)	2.8 (L)	mmol/L	3.5–5.5
Chloride	85 (L)	95	103	103	93 (L)	96	mmol/L	95–110
Urea	4.4	4.5	4.2	5.2	4.8	5.8	mmol/L	3.0–8.0
Creatinine	73	61	59	63	60	78	µmol/L	45–85
eGFR	78 (L)	>90	>90	>90	>90	72	mL/min/1.73 m^2^	>89
Bicarbonate	28	27	25	25	22	25	mmol/L	20–32
Total bilirubin	19 (H)	-	-	-	-	21 (H)	µmol/L	3–15
ALP	50	-	-	-	-	50	U/L	30–115
GGT	22	-	-	-	-	30	U/L	5–35
ALT	14	-	-	-	-	21	U/L	5–30
Total protein	65	-	-	-	-	60 (L)	g/L	63–80
Albumin	39	-	31 (L)	30 (L)	34	34	g/L	33–44
Globulin	26	-	-	-	-	26	g/L	26–41
Total calcium	2.35	-	2.14 (L)	2.19	2.20	2.19	mmol/L	2.15–2.55
Corrected calcium	2.37	-	2.32	2.39	2.32	2.31	mmol/L	2.20–2.60
Phosphate	1.02	-	0.87	1.12	1.34	1.09	mmol/L	0.8–1.5
Magnesium	0.83	-	0.78	0.76	0.72	1.03	mmol/L	0.70–1.05
Haemoglobin	141	122	118	112 (L)	137	143	g/L	115–165
HCT	0.38	0.35 (L)	0.34 (L)	0.33 (L)	0.40	0.42	L/L	0.36–0.47
MCV	84	86	90	90	90	88	n/L	80–100
WBC	1.3 (L)	0.9 (L)	1.0 (L)	1.1 (L)	1.4 (L)	3.7 (L)	n/L	4.0–11.0
Neutrophils	0.2 (L)	0.2 (L)	0.2 (L)	0.1 (L)	0.2 (L)	1.2 (L)	n/L	2.0–7.5
Lymphocytes	0.5 (L)	0.3 (L)	0.3 (L)	0.5 (L)	0.6 (L)	0.8 (L)	n/L	1.0–4.0
Monocytes	0.6	0.5	0.6	0.6	0.6	1.7 (H)	n/L	0.2–1.0
Eosinophils	<0.1	<0.1	<0.1	<0.1	<0.1	<0.1	n/L	<0.5
Basophils	< 0.1	<0.1	<0.1	<0.1	<0.1	<0.1	n/L	<0.3
Platelets	233	195	156	131 (L)	113 (L)	86 (L)	n/L	150–400
nRBC	-	-	-	2.7 (H)	6.5 (H)	1.6 (H)	/100 WBCs	<1.0
Troponin	25 (H)	-	-	-	-	19 (H)	ng/L	<16
CRP	20 (H)	16 (H)	8 (H)	4	14 (H)	27 (H)	mg/L	<5

The remaining blood tests were normal, including nil growth in blood cultures (Table 2). The respiratory viral swab was positive for rhinovirus. Urinary antigens for *Streptococcus pneumoniae* and HIV, CMV, and syphilis serology were negative (Table 3). The patient's hypokalaemia was treated accordingly with intravenous potassium replacement, and with her neutropenia, her mycophenolate and upadacitinib were withheld in the context of a potential meningitis. Initial lumbar puncture opening pressures were not recorded; however, the cerebrospinal fluid showed protein levels of 735 (normal: 150-450 mg/L), glucose of 1.3 (normal: 2.2-3.9 mmol/L), polymorphs of 18 (normal: <1x10^6^/L), lymphocytes/monocytes of 7 (normal: <5x10^6^), and erythrocytes of 136 (normal: <1x10^6^/L), and subsequently a positive culture of *Cryptococcus neoformans* with an antibody titre of 1:1280 and a positive India ink stain (Tables 3, 4).

**Table 2:** Blood Investigations - Other H: abnormally high result, L: abnormally low result, INR: international normalised ratio, APTT: activated partial thromboplastin time, HIV: human immunodeficiency virus

Parameters	Day 1 (22/5/25)	Day 2 (23/5/25)	Day 3 (24/5/25)	Units	Reference Range
INR	0.9	-	1.0	-	0.9–1.2
APTT	27	-	23	Seconds	25–35
Fibrinogen	4.1	-	2.5	g/L	2.0–4.5
Thrombin time	20	-	20	Seconds	15–22
Syphilis antibody	-	Non-reactive	-	-	-
Blood culture	Nil growth	-	-	-	-
HIV ½ antigen/antibody	-	Not detected	-	-	-

**Table 3:** Swabs and Urine (H): abnormally high result, (L): abnormally low result, COVID: Coronavirus disease 2019, RSV: respiratory syncytial virus, MRSA: methicillin-resistant *Staphylococcus aureus*, VRE: vancomycin-resistant enterococci, CPO: carbapenemase-producing organisms

Parameters	Day 1 (22/5/25)	Day 2 (23/5/25)	Day 5 (26/5/25)	Day 6 (27/5/25)
COVID/Influenza A/Influenza B/RSV swab	Negative	-	-	-
*Bordetella pertussis*/*Bordetella parapertussis*/*Mycoplasma pneumoniae*/*Chlamydia pneumoniae* swab	Negative	-	-	-
Rhinovirus/Parainfluenza/Human Metapneumovirus/Adenovirus swab	Rhinovirus positive	-	-	-
Urine	-	*S. pneumoniae* antigen not detected. Urine MCS: Glucose Nil, specific gravity 1.046, Protein +, Ketones +, Blood nil, Nitrite nil, pH 7 (L), Leucocytes 13 x 10^6^/L, Erythrocytes < 4 x 10^6^, Squamous epithelial cells 64 x 10^6^ (H), no bacterial growth	Urine MCS: Glucose Nil, Specific gravity 1.012, Protein Nil Ketones Trace, Blood +, Nitrite Nil, pH 8, Leucocytes < 3, Erythrocytes 30 (H), squamous epithelial cells < 5, no bacterial growth	-
MRSA swab (nose, throat, groin)	-	-	-	Negative
VRE swab (rectum)	-	-	-	VRE (*Enterococcus faecium*). vanB Gene Detected
CPO – Carbapenemase (rectum)	-	-	-	Negative

**Table 4:** Lumbar Puncture and Cerebrospinal Fluid Investigations Tube 1: First 3–5 mL of cerebrospinal fluid collected Tube 2: Second 3–5 mL of cerebrospinal fluid collected Tube 3: Third 3–5 mL of cerebrospinal fluid collected H: abnormally high result, L: abnormally low result, CSF: cerebrospinal fluid, Lymph/Mono: lymphocytes/monocytes

Parameters	Day 1 (22/5/25)	Day 3 (24/5/25)	Day 5 (26/5/25)	Day 6 (27/5/25) On transfer to tertiary hospital
Opening pressures	-	21 cm H_2_O	>35 cm H_2_O (H)	-
Tube 1	Protein 735 mg/L (H) Glucose 1.3 mmol/L (L) Polymorphs 18 x 10^6^/L (H) Lymph/Mono 7 x 10^6^/L (H) Erythrocytes 136 x 10^6^/L (H)	Protein 580 mg/L (H) Glucose 2.5 mmol/L Polymorphs 20 x 10^6^/L (H) Lymph/Mono 80 x 10^6^/L (H) Erythrocytes 200 x 10^6^/L (H)	Protein 1314 mg/L (H) Glucose 1.3 mmol/L (L) Polymorphs 2 x 10^6^/L (H) Lymph/Mono 92 x 10^6^/L (H) Erythrocytes 0 x 10^6^/L	Protein 1.04 g/L (H) Glucose 1.04 mmol/L (L) Polymorphs < 1 x 10^6^/L Lymphocytes 89 x 10^6^/L (H) Erythrocytes 6 x 10^6^/L (H) Total Leucocytes 89 x 10^6^/L (H)
Tube 2	Polymorphs 17 (H) Lymph/Mono 5 (H) Erythrocytes 119 (H)	Polymorphs 60 (H) Lymph/Mono 70 (H) Erythrocytes 50 (H)	-	-
Tube 3	Polymorphs 13 (H) Lymph/Mono 7 (H) Erythrocytes 96 (H)	Polymorphs 20 (H) Lymph/Mono 90 (H) Erythrocytes 230 (H)	-	-
CSF Culture	*Cryptococcus neoformans* CSF viral multiplex –ve (HSV, VZV, Enterovirus, Parechovirus, *L. monocytogenes* DNA). CMV negative	Cryptococcus neoformans	No growth after 48 hours of microscopic incubation: Smear is moderately cellular and shows prominent small mature lymphocytes and histiocytes. No definite cryptococcal organisms are seen. Diagnosis: cerebrospinal fluid: Negative for malignancy	*Cryptococcus neoformans* India Ink Stain - encapsulated yeasts seen CSF Cytospin: Numerous lymphocytes, including large reactive forms, and many monocytes. Rare erythrocytes seen. Please correlate with ancillary tests.
CSF Antigen	Cryptococcal antigen: detected titre 1:1280	-	-	-

**Figure 1:**
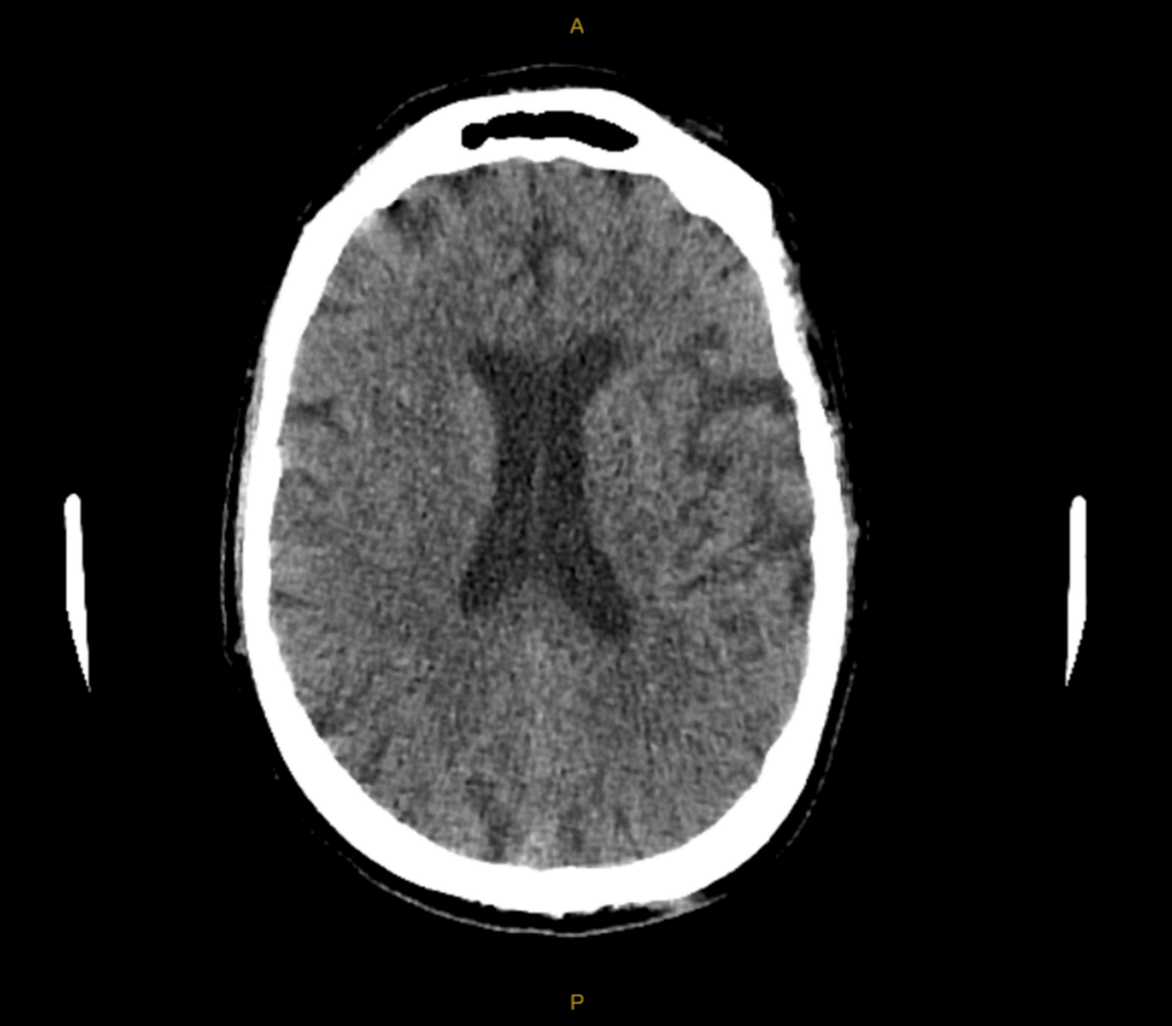
CT Brain Scan With Contrast Day 1 - Normal Study CT: computerised tomography

**Figure 2:**
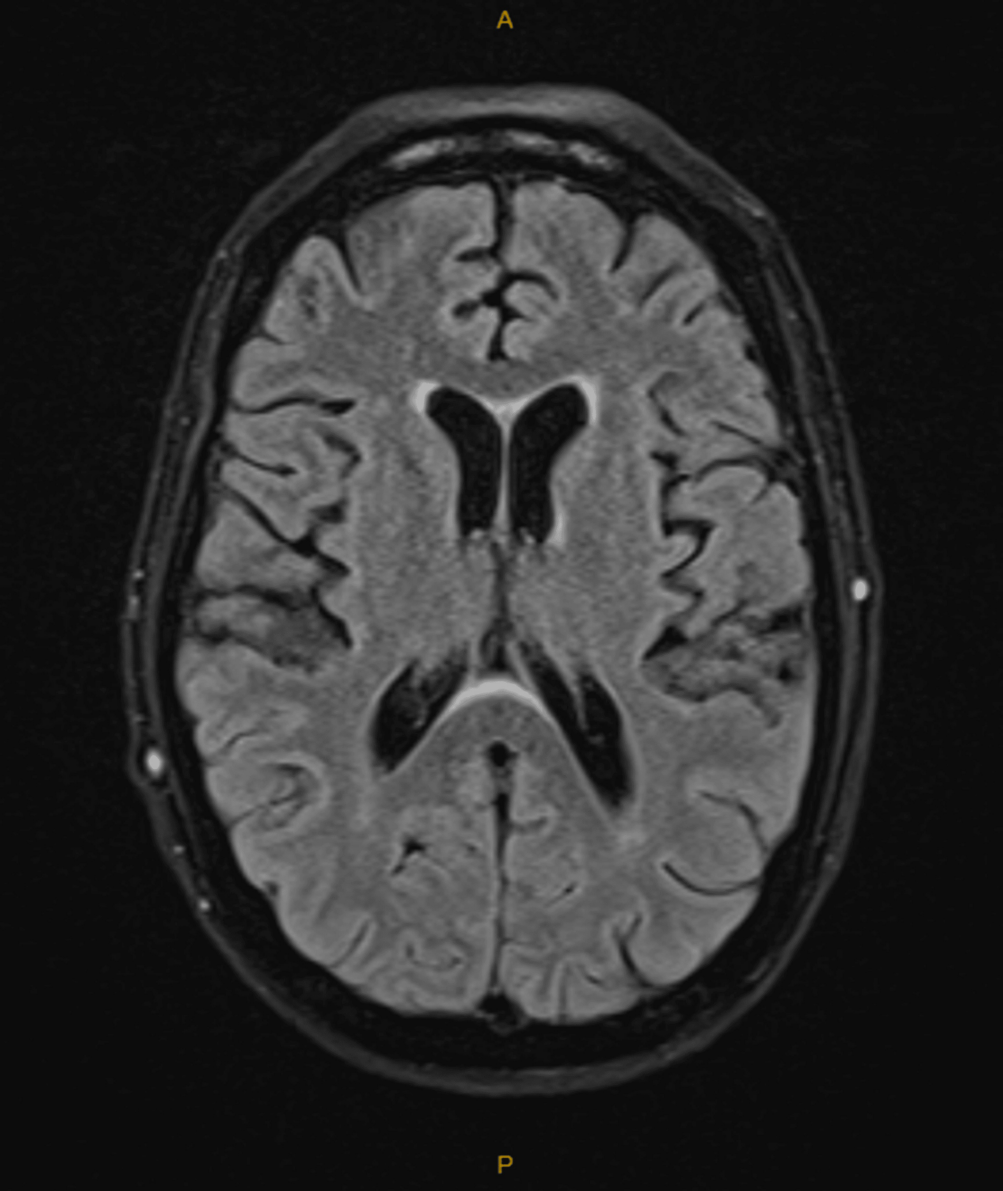
MRI Brain T2 FLAIR With Contrast Day 2 - Normal Study MRI: magnetic resonance imaging, T2 FLAIR: T2-weighted fluid-attenuated inversion recovery

The brain CT showed no intracranial abnormalities, and the brain MRI reported no acute infarcts, no space-occupying lesions, and no evidence of meningitis or encephalitis (Figures 1, 2).

The patient was commenced on amphotericin B 250 mg daily and oral flucytosine 2000 mg six-hourly. A chest X-ray was completed, which did not show any pulmonary disease. A repeat lumbar puncture done two days after the initial positive CSF culture showed an opening pressure of 21 cmH₂O, a protein of 580 mg/L, glucose of 2.5 mmol/L, polymorphs of 20x10^6^/L, lymphocytes/monocytes of 80x10^6^/L and was culture positive for *Cryptococcus neoformans* (Table 4). Two days later, an additional lumbar puncture was done due to increased somnolence: this demonstrated a significantly increased opening pressure of >35 cmH₂O, with protein of 1314 mg/L, glucose of 1.3 mmol/L, polymorphs of 2x10^6^/L, and lymphocytes/monocytes of 92x10^6^/L (Table 4).

**Figure 3:**
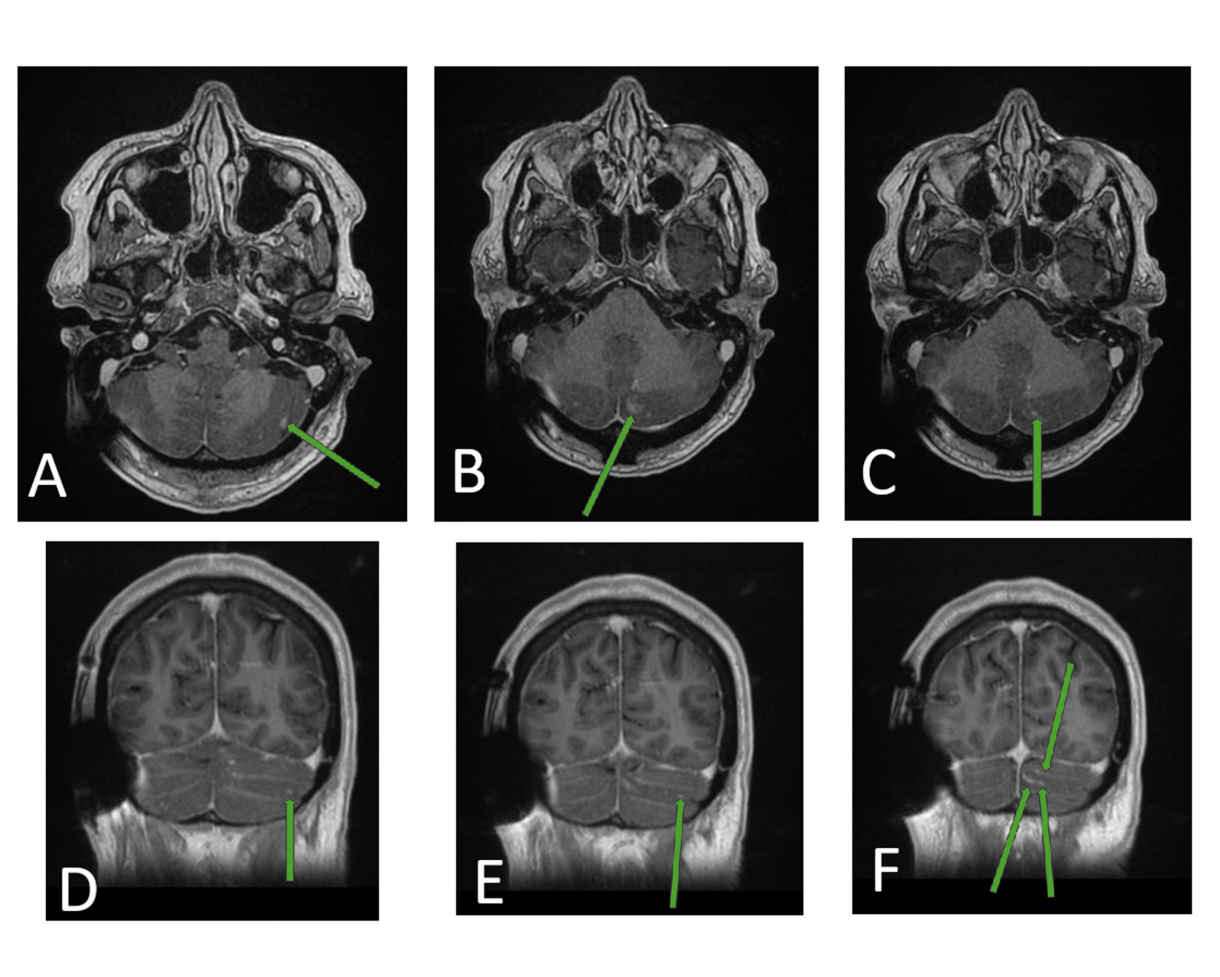
MRI Brain and Spine T1 With Contrast Showing Small Volume Leptomeningeal Enhancement in the Posterior Fossa (A–F) A–C: Axial MRI (T1 with contrast) with progressively caudal slices showing small volume leptomeningeal enhancement in the posterior fossa D–F: Coronal MRI (T1 with contrast) with progressively posterior slices showing small volume leptomeningeal enhancement in the posterior fossa MRI: magnetic resonance imaging

With a significant increase in opening CSF pressure in 48 hours, risk of neurodeterioration, and a likely requirement of daily lumbar punctures in a rural hospital, she was transferred to a tertiary hospital under the Neurosurgery team for insertion of a lumbar drain, then subsequently an insertion of a ventriculoperitoneal shunt and completion of her course of antifungals. A subsequent MRI of the spine on Day 23 of admission showed small volume leptomeningeal enhancement in the posterior fossa consistent with known meningitis (Figure 3).

Flucytosine was withheld prior to two weeks’ duration due to pancytopaenia, and amphotericin B was continued for two weeks before stepping down to fluconazole 400 mg daily for eight weeks and reduced to 200 mg daily for one year. Ophthalmology reviewed the patient towards the end of her hospital stay and visualised mild bilateral papilloedema, likely secondary to her cryptococcal infection, but no recurrence of HSV keratitis or CMV retinitis. The patient’s discharge plan was an ongoing follow-up with Neurology, Infectious Diseases, and Ophthalmology. Two months post-discharge, she was recommenced on upadacitinib by her rheumatologist with no reported relapse as of seven months post-discharge. All other immunosuppression, including mycophenolate, has been withheld, and she will have ongoing Dermatology review for her EBA.

## Discussion

General risk factors for non-HIV individuals acquiring CM include corticosteroid treatment, solid organ transplantation, liver cirrhosis, cancer, monoclonal antibodies, sarcoidosis, and tyrosine kinase inhibitors [2,4-7]. At the time of writing, there is no specific literature on EBA patients who develop CM due to immunosuppression. A recent retrospective case-control study described the characteristics of 20 patients with RA who developed cryptococcal infections, all of whom were on long-term corticosteroids (3.9±3.3 years), 65.0% were over 60 years of age, and 65.0% were female [9]. The study identified specific risk factors, including corticosteroids, chronic kidney disease, and adalimumab, none of which our patient was taking at the time of presentation [9]. Some patients were concurrently taking prednisolone with DMARDs and biologics such as anti-TNF and etanercept, though it was not specified what each individual patient was taking [9]. There was also one case report of a patient taking prednisolone and etanercept simultaneously at the time of developing CM [10].

The pathogenesis of CM is poorly understood and can either progress post-primary infection (pulmonary cryptococcus) rapidly or reside in phagocytes for up to 110 months (approximately nine years) in *C. neoformans* [5]. As our patient had been on both MMF and upadacitinib for the last five years and had previously undergone extensive immunosuppression, perhaps it was the recent primary respiratory illness that preceded our patient’s onset of CM, rather than an infection that had been dormant for years in the context of immunosuppression.

One limitation to be acknowledged was that the lumbar puncture opening pressure was not recorded at the patient's initial presentation. It is therefore unknown whether the patient presented with high opening pressures or whether this had developed during her hospital stay.

Some cases of CM have been reported in patients taking mycophenolate, and evidence from zebrafish models suggests that the pathway affects macrophages [11]. JAK inhibitors dampen multiple cytokines, including IFN-γ and GM-CSF, essential cytokines for the clearance of cryptococcal infections [8]. A 2024 literature review identified three published reports of patients on ruxolitinib, a JAK inhibitor, who were not on MMF and who subsequently developed CM [8]. Upadacitinib was approved by the FDA in August 2019, and there are warnings associated with pulmonary cryptococcosis and bacterial meningitis, but not specifically CM [12]. As of February 2026, there are no publications related to CM in which a patient is simultaneously on upadacitinib and mycophenolate.

Some studies suggest that the in-hospital mortality of HIV-negative people with CM is higher than that of those with HIV [4]. It is therefore important to raise awareness of the risk of CM in HIV-negative individuals on multiple immunosuppressants or novel therapies, such as JAK inhibitors, which are used to treat a wide variety of autoimmune conditions.

## Conclusions

The risk of occurrence of CM in HIV-negative patients is not fully known and brings awareness to a potentially vulnerable population group in immunosuppressed individuals who are not transplant recipients, especially those taking new targeted therapies such as JAK-2 inhibitors or who are on more than one immunosuppressive therapy. 

This case emphasises the need for heightened clinical awareness of CM and to consider individuals who may be taking targeted or multiple immunosuppression therapies. Future research should aim to elucidate the risk of CM for HIV-negative individuals taking older immunosuppressants such as MMF, newer biologics such as JAK inhibitors, or those taking more than one immunosuppressive medication and assess effective screening and treatment strategies for these complex presentations.
